# Phylogenetic diversity drives soil multifunctionality in arid montane forest-grassland transition zone

**DOI:** 10.3389/fpls.2024.1344948

**Published:** 2024-02-12

**Authors:** Xiaofei Wang, Lu Gong, Yan Luo, Zhaolong Ding, Qian Guo, Xiaochen Li, Xinyu Ma

**Affiliations:** ^1^ College of Ecology and Environment, Xinjiang University, Urumqi, China; ^2^ Key Laboratory of Oasis Ecology, Ministry of Education, Urumqi, China

**Keywords:** taxonomic diversity, functional diversity, phylogenetic diversity, soil multifunctionality, forest-grassland transition zones

## Abstract

Exploring plant diversity and ecosystem functioning in different dimensions is crucial to preserve ecological balance and advance ecosystem conservation efforts. Ecosystem transition zones serve as vital connectors linking two distinct ecosystems, yet the impact of various aspects of plant diversity (including taxonomic, functional, and phylogenetic diversity) on soil multifunctionality in these zones remains to be clarified. This study focuses on the forest-grassland transition zone in the mountains on the northern slopes of the Tianshan Mountains, and investigates vegetation and soil characteristics from forest ecosystems to grassland ecosystems to characterize plant diversity and soil functioning, as well as the driving role of plant diversity in different dimensions. In the montane forest-grassland transition zone, urease (URE) and total nitrogen (TN) play a major role in regulating plant diversity by affecting the soil nutrient cycle. Phylogenetic diversity was found to be the strongest driver of soil multifunctionality, followed by functional diversity, while taxonomic diversity was the least important driver. Diverse species were shown to play an important role in maintaining soil multifunctionality in the transition zone, especially distantly related species with high phylogeny. The study of multidimensional plant diversity and soil multifunctionality in the montane forest-grassland transition zone can help to balance the relationship between these two elements, which is crucial in areas where the ecosystem overlaps, and the application of the findings can support sustainable development in these regions.

## 1 Introduction

In ecosystem transition zones, changes in various mechanisms, including information, energy, and matter, affect ecological patterns and processes, and thus ecosystem function. The transition from forest to grassland ecosystems involves changes in biotic and abiotic conditions, such as temperature, solar radiation intensity, and litter input, which strongly affect species composition and cause rapid changes in functional structure at spatial and temporal scales (Kim et al., 2017; Erdős et al., 2018; Cao et al., 2019; Rippel et al., 2020). The forest-grassland transition zone marks the boundary between two distinct ecosystems. Most research on ecosystem edges has been conducted from the interior towards the edge. For instance, in forest ecosystems, the microclimate at the edges is warmer and drier, which is likely due to differences in canopy structure and plant functional traits. This microclimate accelerates the decomposition of litter, resulting in significantly lower soil carbon content than in the forest interior (Ordway and Asner, 2020; Rippel et al., 2020). Leaf mass was significantly decreased, while the foliar nitrogen and phosphorus content showed an increase in forest edge plants (Ma et al., 2017). The study of ecosystems remains incomplete due to insufficient research on the bilateral edge effect approach in the transition zone between forest and grassland ecosystems (Erdős et al., 2018).

Further research is necessary to fully comprehend the relationship between plant diversity and ecosystem functioning in the transition zone between forest and grassland. Previous studies have overlooked the potential of ecosystems to provide multiple functions, leading to a deficiency in extensive exploration of ecosystem functions and individual biodiversity (van der Plas, 2019). Differences in the impact of various dimensions of plant diversity (taxonomic, functional, and phylogenetic diversity) on ecosystem functioning (Steudel et al., 2016; Liu et al., 2022; Scherer-Lorenzen et al., 2022). Changes in taxonomic diversity have the potential to affect the way species are distributed among resources during the growth cycle, as well as impact species interactions, including competition, facilitation, and symbiosis, and the presence of keystone and dominant species, all of which can further influence ecosystem functioning (Stavert et al., 2019). Increased taxonomic diversity and positive species interactions contribute to enhanced ecosystem biomass (Pöpperl and Seidl, 2021). Diversified plants and interspecific competition for ecological niches can improve soil nutrient uptake and utilization, increase above- and below-ground litter, promote nutrient cycling, and return nutrients (Santonja et al., 2017). The impact of functional diversity on ecosystem functioning is frequently achieved through mechanisms like resource acquisition and plant-soil feedback. High functional diversity implies a high degree of complementarity in resource utilization among species. Differences in plant functional traits affect nutrient uptake. Plants with low specific leaf area, leaf nitrogen, and phosphorus content, and high leaf dry weight and leaf thickness generally have a greater capacity for soil nutrient uptake (Jager et al., 2015). Phylogenetic diversity characterizes the affinities and functional differentiation of species. Different species exhibit varying physiological characteristics and ecological roles based on their phylogenetic relationships, resulting in variability in function. This variability can be used as a proxy for unmeasured functional diversity to further investigate the effects of plant diversity on ecosystem functioning (Cadotte, 2013). Communities consisting of species with different ecological strategies are crucial for maintaining ecosystem functions. Communities with high functional and phylogenetic diversity exhibit greater differences in leaf traits, root secretions, and root turnover rates among species, as well as a greater diversity of litter, which promotes soil nutrient cycling (Le Bagousse-Pinguet et al., 2019; Valencia et al., 2022). According to the multidimensional study, it was discovered that plant diversity and multifunctionality in arid ecosystems exhibit a close relationship, despite various disruptions (Le Bagousse-Pinguet et al., 2019). Therefore, examining the correlation between diversity and soil multifunctionality from three perspectives (taxonomic diversity, functional diversity and phylogenetic diversity) is crucial for comprehending the mechanism behind plant diversity preservation and predicting the ecological outcomes of plant diversity depletion. This understanding can be crucial for decision-making on sustainability.

The Tianshan Mountains contain temperate coniferous forests dominated by Picea schrenkiana, typical arid mountain ecosystems whose distribution is influenced by climate and topography, resulting in uneven cover (Zhu et al., 2022). The transition zone between the forest and grassland ecosystems is rich in vegetation types and undergoes rapid changes. It has complex ecosystem structures, functions, and ecological processes, and is sensitive and fragile to environmental changes. Due to climate warming and intensified drought, the transition zone is moving towards forests, which puts some species in danger of disappearing (Cheng et al., 2020). To maintain ecosystem function and plant diversity in the arid zone, it is necessary to clarify the characteristics and interrelationships between plant diversity and soil function in the transition zone. This study examines the characteristics of vegetation and soil in the forest-grassland ecosystem transition zone on the northern slopes of the Tianshan Mountains. The aim is to address two scientific questions: 1. To characterize changes in plant diversity and soil multifunctionality in the forest-grassland transition zone; 2. To explore the role of plant diversity in driving soil multifunctionality in the transition zone and to determine which dimension of diversity plays a stronger role. The results of this study will provide valuable reference for vegetation conservation and regional ecological stability in the ecological transition zone.

## 2 Materials and methods

### 2.1 Study site

The study site is located in the middle part of the northern slope of the Tianshan Mountains in Xinjiang (87°28.272′ - 87°28.908′ W, 43°26.130′ - 43°26.130′ N). The study site experiences a temperate continental climate with an average annual temperature ranging between 2-3°C, 1134.1 mm of annual rainfall, 544.0 mm evaporation rate, and an average annual relative humidity of 65%. There is rich vegetation in the forest-grassland transition zone, among which the tree plants are mainly Picea schrenkiana. Shrub plants include Berberis atrocarpa and Rosa platyacantha. The herbs are mainly Carex turkestanica, Alchemilla japonica, Aegopodium alpestre, Galium odoratum, etc (**Supplementary Table 1**). The soil is a gray-brown forest soil with a high degree of soil development and a thick humus layer.

### 2.2 Experimental design and sample collection

**Figure 1:**
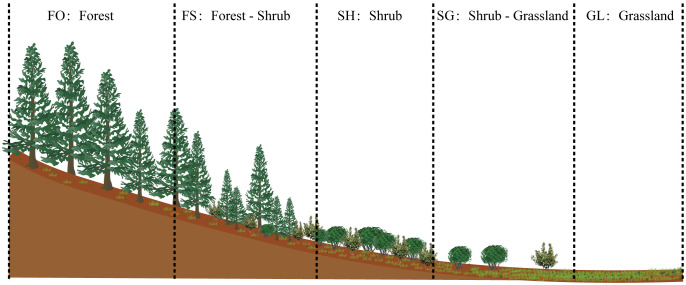
Sample map of the study site.

Based on the vegetation characteristics of the forest-grassland ecosystem transition zone in the Tianshan Mountains, we established five sampling gradients during the transition from forest to grassland (**Figure 1**). These gradients included Forests (FO), Forest-Shrub (FS), Shrubs (SH), Shrub-Grassland (SG), and Grassland (GL). In each gradient, we selected three plots, resulting in a total of 15 samples. To conduct surveys on the vegetative species count and traits, document the name and quantity of each species in the samples, and gather soil samples from 0-15 cm at three points along the diagonal within the sample plots. Mix the soil samples thoroughly.

### 2.3 Experimental measurements

Determination of soil samples: Organic carbon(SOC) was determined by Potassium dichromate oxidation method; Invertin(INV) was determined by 3, 5-dinitrosalicylic acid colorimetric method; total nitrogen(TN)was determined by Kjeldahl nitrogen determination; alkaliolytic diffusion method was used to determine alkaliolytic nitrogen(AN); nitrate nitrogen(NO_3_^–^–N) was determined by phenol disulfonic acid colorimetric assay; ammonium nitrogen(NH_4_^+^-N) was determined by leaching and indophenol blue colorimetric assay; urease was determined by sodium phenol-sodium hypochlorite colorimetric assay; total phosphorus was determined by HCLO_4_-H_2_SO_4_; available phosphorus(AP) was determined by 0.5 mol·L^-1^ NaHCO_3_ molybdenum antimony antimony colorimetric method; disodium phosphate colorimetric method for neutral phosphatase(NP). Plant height (H) was measured and healthy plant leaves were collected in different orientations, plant height (H), leaf length (LL), leaf width (LW), leaf thickness (LT), leaf dry weight (LW) were measured and leaf area (LA) and specific leaf area index (SLA) were calculated. Leaf samples were collected to determine leaf carbon content (LC) by potassium dichromate oxidation, leaf nitrogen content (LN) by nesslerization, and leaf phosphorus content (LP) by vanadium-molybdenum yellow colorimetric method.

### 2.4 Metrics of multidimensional diversity

Taxonomic, functional, and phylogenetic diversity indicators were calculated by assessing species counts and measuring plant functional traits through utilization of R 4.3.1.

Taxonomic diversity reflects the interplay among species and their environment, as well as the stability and complexity of ecosystem structure. These indices consider both the number and evenness of species present in the ecosystem. Several indices have been used to assess taxonomic diversity, including Margalef index, Shannon-Weiner index, Simpson index (Simpson, 1949), and Pielou index.

Functional diversity refers to the variations in functional traits between species within a community and their response to environmental stressors or disruptions. Using a set of 10 plant traits (**Supplementary Figure 2**) including H, LL, LW, LT, LDM, LA, SLA, and LC, LN, LP, we calculated the RaoQ quadratic entropy coefficient with the ‘FD’ package to determine functional diversity, functional dispersion (FRis), functional convergence (FDiv), and functional evenness (FEve) indices. These indices offer a representation of functional diversity based on the functional traits of plants.

For phylogenetic diversity, species names and classifications were first determined by the APG III classification system, a phylogenetic tree was constructed using the ‘rtrees’ package, and then based on the phylogenetic tree, the phylogenetic diversity was quantified by calculating Faith’s PD (Faith, 1992), the mean nearest taxon distance (MNTD) and nearest-taxon-index (NTI) as well as mean pairwise distances (MPD) using the ‘picante’ package (Webb et al., 2008; Kembel et al., 2010). The Faith’PD represents the least total branch length on the phylogenetic tree. The MNTD is the weighted average of the phylogenetic distances between each taxonomic unit and its closest relative in the community. The NTI measures the standardized effect size of the MNTD, and the MPD represents the weighted average of the average phylogenetic distances between taxonomic units in the community.

### 2.5 Measuring soil multifunctionality

Soil function was characterized by measuring 10 soil nutrient indicators which are associated with ecosystem processes. For the carbon cycle, we measured organic carbon and invertase; for the nitrogen cycle, we measured total nitrogen, available nitrogen, nitrate nitrogen, ammonium nitrogen, and urease; and for the phosphorus cycle, we measured total phosphorus, available phosphorus, and neutral phosphatase. Quantification of overall soil multifunctionality using the mean value approach through R 4.3.1. (Wang et al., 2021). The functional values underwent transformation, followed by normalization of the data, and ultimately, the mean for each functional value was computed, which resulted in calculating the soil multifunctionality. The multifunctionality index SMF is calculated as.


$$\text{SMF}=\frac{1}{F}\sum_{i=1}^{F}g(r_{i}(f_{i}))$$

Where *F* represents the number of functions measured, *f_i_* represents the value of function *i*, *r_i_* is a mathematical function that converts *f_i_* to a positive value, and *g* represents the normalization of the measured values.

### 2.6 Statistical analysis

Plant diversity and soil characteristics were investigated in ecosystem transition zones through one-way ANOVA to determine statistical differences. The study also utilized the least significant difference (LSD) multiple comparisons and calculated coefficient of variation (CV) with SPSS 26.0 software (SPSS, IBM, United States). The correlation between the two variables was examined through redundancy analysis (RDA) and Pearson’s correlation, by using Canoco 5.0 software and R 4.31. The study investigated the correlation between plant diversity and soil multifunctionality, utilizing Partial Least Squares Path Modeling (PLS-PM) with the ‘plspm’ package for R 4.3.1. The three dimensions of soil multifunctionality were examined in terms of their influence by the model. The loading coefficient was utilized to exclude indicators with less than 0.7 score to enhance the model’s efficacy. The primary drivers of multifunctionality in plant diversity indicators within ecosystems were identified through random forest analysis using the ‘randomForest’ package of R 4.31.

## 3 Results

### 3.1 Soil characteristics of the forest-grassland transition zone

**Figure 2:**
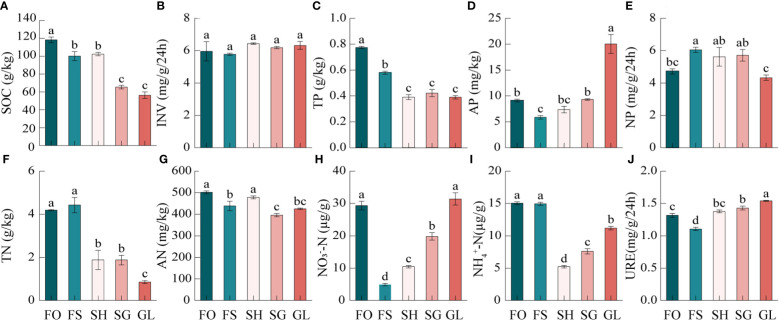
Characteristics of soil nutrients in the forest-grassland ecosystem transition zone. SOC, organic carbon; INV, invertase; TN, total nitrogen; AN, available nitrogen; NO_3_^–^–N, nitrate nitrogen; NH_4^+^_–N, ammonium nitrogen; URE, urease; TP, total phosphorus; AP, available phosphorus; NP, neutral phosphatase. FO, Forest; FS, Forest-Shrub; SH, Shrub; GL, Grassland. Different lowercase letters indicate significant (*p* < 0.05) differences between soils of different gradients.

As shown in **Figure 2**, during the transition from forest to grassland, the soil nutrient properties exhibited significant variations. SOC and TP levels were the highest in FO and the lowest in GL, respectively. The TN content was not significantly different from FO, although it was highest in FS. Consequently, the trend of SOC, TN, and TP during the FO to GL transition demonstrated an overall pattern of FO>SH>GL. Among the quick-acting nutrients, GL had the highest AP while FS had the lowest AP. AN demonstrated a significant gradient with the highest levels in FO and the lowest levels in SG. The trend of NO_3_^–^–N was higher in FO and gradually increased after the lowest levels observed in FS. The change trend of NH_4_^+^-N was similar to NO_3_^–^–N but reached its lowest point in SH. NH_4_^+^-N was also similar to NO_3_^–^–N, but had its lowest concentration in SH. Of the three enzymes associated with the carbon, nitrogen, and phosphorus cycles, respectively, INV was not significantly different in the transition zone. In contrast, NP was significantly higher in FS than in FO, significantly lower in GL than in FS, SH, and SG, but not significantly different from FO. GL had the lowest levels, while FS had the highest. URE, on the other hand, was significantly higher in GL and significantly lower in FS. The variability of soil nutrients and enzyme activities in the transition zone was primarily weak to moderate. INV and AN exhibited a coefficient of variation (CV) of<10% in this area, while the other nutrients fell within a range of 11.87 to 60.19%. The order of variation magnitude was as follows: NO_3_^–^–N>TN>AP>NH_4_^+^-N>TP>SOC>NP>URE>AN>INV (**Supplementary Table 2**).

### 3.2 Characterizing changes in plant diversity in the forest-grassland transition zone

**Figure 3:**
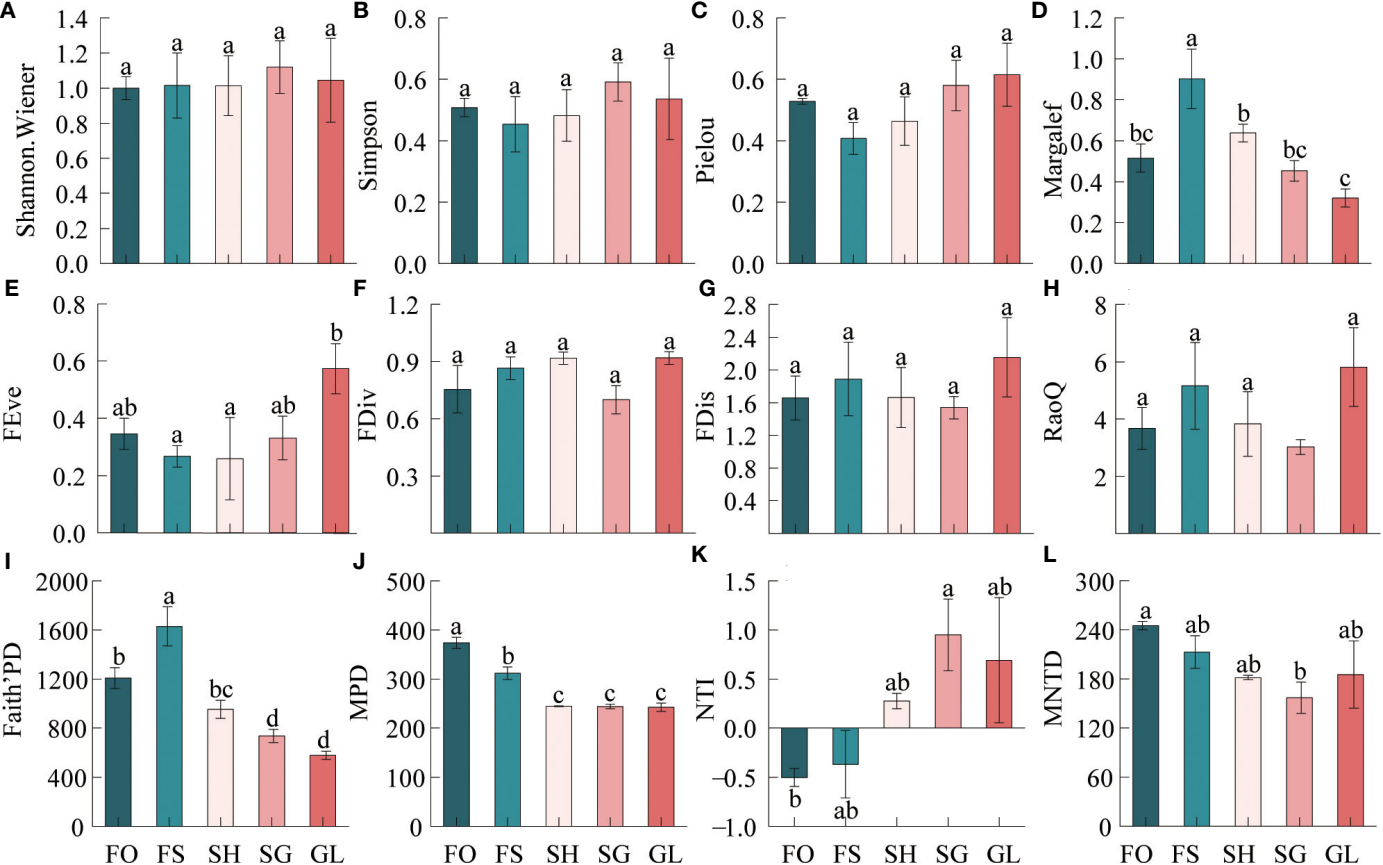
Characteristics of biodiversity in the forest-grassland ecosystem transition zone. FRis, functional dispersion; FDiv, functional convergence; FEve, functional evenness; RaoQ, the RaoQ quadratic entropy coefficient. MPD, mean pairwise distances; NTI, nearest-taxon-index; MNTD, mean nearest taxon distance. FO, Forest; FS, Forest-Shrub; SH, Shrub; GL, Grassland. Different lowercase letters indicate significant (*p* < 0.05) differences in plant diversity indices at different gradients.

The **Figure 3** indicates that plant diversity exhibits varying change characteristics during the transition from forest to grassland. Regarding taxonomic diversity, the Margalef index demonstrates significant variability during the transition, with a trend of increasing and then decreasing in the order of FS>SH>FO>SG>GL.

Functional diversity was highest in GL, with all four indices reaching their highest values, while FEve was the lowest in SH. FDiv, FDis and RaoQ were all the lowest in SG. The order for the size of the FEve index was GL>FO>SG>FS. For the FDiv index, it was GL>SH>FS>FO>SG. The FDis index varied in the same way as the RaoQ index, with GL>FS>SH>FO>SG. The community-weighted means of plant traits displayed significant variation across the gradient. H, LL, LW, and SLA all exhibited a decreasing trend, while LT showed the opposite trend, becoming progressively thicker in the transition zone. Leaf carbon content was consistently higher throughout the gradient, while nitrogen and phosphorus content were higher in forests and grasslands (**Supplementary Figure 2**).

In terms of phylogenetic diversity, Faith’s PD index exhibited a gradual decrease from FO to FS, with FS being greater than FO, SH, SG, and GL. The MPD index also saw a gradual decrease from FO to GL, and FO was notably higher than the other samples. The NTI index, on the other hand, showed a trend of SG>GL>SH>FS>FO. Specifically, FO was greater than FS, SH, SG, and GL. At FO and FS, SG was negatively correlated, indicating phylogenetic dispersion. Additionally, SG was the highest and significantly greater than FO. Positive values at SH, SG, and GL indicate phylogenetic clustering. The NTI indices for all sampled sites ranged from -1.96 to 1.96. The MNTD index decreased from FO to SG, then increased at GL, with FO>FS>GL>SH>SG; SG was significantly lower than FO. The CV value of the Shannon Wiener index of taxonomic diversity indicated weak variability at 4.66%. The variance was weak. The coefficient of variation (CV) for NTI, an index measuring phylogenetic diversity, was 304.27%, indicating strong variation. The remaining indices had moderate CV values. The order of strength of variation was, taxonomic diversity: Margalef>Pielou>Simpson>Shannon.Wiener; functional diversity: FEve>RaoQ>FDis>FDiv; phylogenetic diversity: NTI>Faith’PD>MPD>MNTD (**Supplementary Table 2**).

### 3.3 Ecological functions in relation to plant diversity

**Figure 4:**
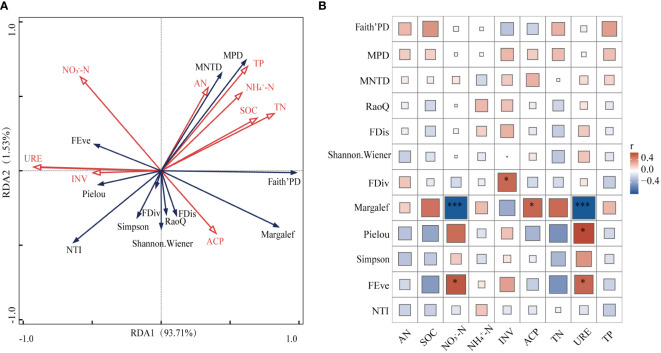
Relationship between soil nutrients and biodiversity. SOC, organic carbon; INV, invertase; TN, total nitrogen; AN, available nitrogen; NO_3_^–^–N, nitrate nitrogen; NH_4^+^_–N, ammonium nitrogen; URE, urease; TP, total phosphorus; NP, neutral phosphatase. FRis, functional dispersion; FDiv, functional convergence; FEve, functional evenness; RaoQ, the RaoQ quadratic entropy coefficient. MPD, mean pairwise distances; NTI, nearest-taxon-index; MNTD, mean nearest taxon distance. * Indicates (*p* < 0.05); *** Indicates (*p* < 0.001).

As shown in **Figure 4**, among the taxonomic diversity, Margalef index had highly significant negative correlations with both NO_3_^–^–N and URE (*p*<0.001) and significant positive correlations with NP (*p*<0.05). The strongest significance was with NO_3_^–^–N and URE (*p*<0.001). pielou was significantly positively correlated with URE (*p*<0.05). In functional diversity, FEve showed significant positive correlation with NO_3_^–^–N and URE (*p*<0.05) and FDiv was significantly positively correlated with INV (*p*<0.05). Phylogenetic indicators were not significantly related to soil indicators.

Based on the results of RDA conducted on 12 indicators of plant diversity and 9 soil factors, it is evident that the first axis has a correlation of 0.9786 and the second axis has a correlation of 0.9548. The interpretation of RDA1 and RDA2 are 93.71% and 1.53%, respectively, and these two axes are able to account for 95.24% of the variance information.

URE and TN were significant factors impacting plant diversity within the transition zone, explaining 79.9% (*p*<0.01) and 5.9% (*p*<0.05), respectively. These two soil nutrients were found to be the primary drivers of plant diversity within the region. It is important to note the influence of these factors on plant diversity in the transition zone.

### 3.4 Drivers of soil multifunctionality

**Figure 5:**
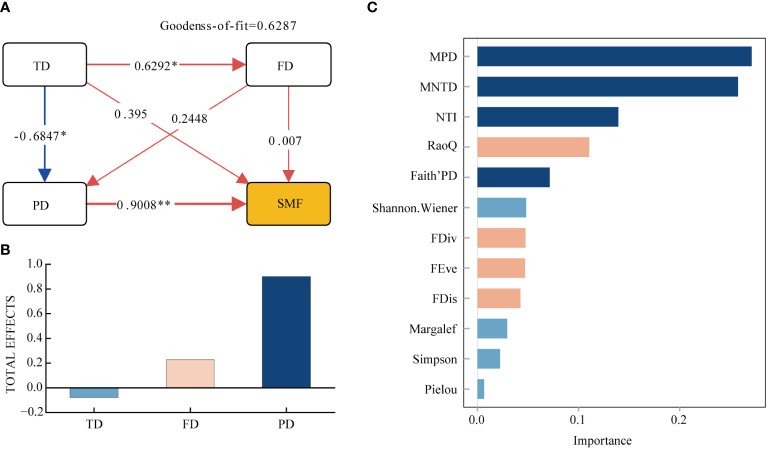
Partial least squares path modeling **(A, B)** and Random Forest Importance Ranking **(C)**. TD, Taxonomic Diversity; FD, Functional Diversity; PD, Phylogenetic diversity; SMF, Soil Multifunctionality. * Indicates (*p* < 0.05); ** Indicates (*p* < 0.01).

Based on the factors that drive soil multifunctionality (SMF), we utilized the partial least squares (PLS) approach to construct a fundamental model that connects taxonomic diversity indicators, functional diversity indicators, and phylogenetic diversity indicators to SMF (**Figures 5A, B**). The model demonstrates that taxonomic, functional, and phylogenetic diversity all positively correlate with SMF. Among them, phylogenetic diversity exhibits the largest and most significant impact, with a direct effect of 0.9008. The most important factors for SMF in terms of diversity were MPD, MNTD, and NTI, according to the Random Forest order of importance (**Figure 5C**). Following these were the total effect of functional diversity on SMF, with a value of 0.2275, and then taxonomic diversity. Nevertheless, taxonomic diversity had an indirect impact on SMF, with a significant positive influence on functional diversity and a significant negative effect on phylogenetic diversity. The results of the random forest analysis indicate that Shannon-Wiener index is the most important factor for soil multifunctionality in terms of taxonomic diversity, followed by Margalef, Simpson, and Pielou indices. For functional diversity, RaoQ was the most important, followed by FDiv, FEve, and FDis. In terms of phylogenetic diversity, MPD, MNPD, and NTI were all more important than both taxonomic and functional diversity indicators.

## 4 Discussion

### 4.1 Characteristics of plant diversity and ecological functions in different dimensions of forest-grassland intertwined zone

Soil nutrients play a critical role in regulating the structure and function of ecosystems and can aid in characterizing nutrient cycling functions. In the transition zone from forest to grassland, there was a gradual decrease in SOC, TN, and TP contents (**Figures 2A, F, C**). These trends indicate a synergistic pattern of change, with significantly higher levels observed in forest compared to other areas. The degree of variation was moderate and in line with previous studies. (Boudjabi and Chenchouni, 2022). Soil nutrients primarily derive from the decomposition of dead roots and plant residue. Picea schrenkiana forest and shrublands are rich in litter material, and coniferous forests are weakly acidic soils with high soil moisture, which promotes SOC accumulation (Zhang et al., 2021). During the transition from forest to grassland, the tall and dense trees gradually transformed into relatively short and sparse shrubs (**Supplementary Table 1**). Additionally, the increase in solar radiation and temperature reduced the retention capacity of carbon and accelerated the decomposition rate of litter material, leading to a consistent decrease in carbon content, which corroborated other scholars’ findings (**Supplementary Figure 1**) (Ma et al., 2017; Rippel et al., 2020). Trees and shrubs have well-developed root systems that produce nitrogen-containing secretions, dead roots, epidermal shedding of root hair tissue, and a large number of inter-root microorganisms and other organic matter that accumulate at the inter-root level and accelerate nitrogen accumulation (**Figure 2F**) (Knelman et al., 2018). The rise in soil and ambient temperatures in grasslands may have caused a reduction in root secretions containing nitrogen and carbon (**Supplementary Figure 1**). This, in turn, affects the diversity and composition of inter-root soil microbes, reduces the expression of functional genes related to soil carbon and nitrogen cycling, and lowers soil carbon and nitrogen content (Yu et al., 2024). TP content decreased gradually during the transition from forest to grassland (**Figure 2C**). It was notably higher in FO and FS regions compared to other areas. This can be attributed to the high forest vegetation cover, fine root biomass, and litter biomass, which increased rainfall retention and soil water-holding capacity of canopy and litter material, thereby reducing runoff and soil phosphorus loss (Fu et al., 2020). AP content significantly increased in the grassland during the transition from forest to grassland, yet decreased in all other scenarios. This occurrence may be attributed to the trees and shrubs storing substantial amounts of phosphorus during the growing season, influencing the soil phosphorus content (Lloyd et al., 2000).

Ecological transition zones frequently exhibit greater taxonomic diversity than surrounding systems. However, intermediate states and low diversity investigations also exist (Lloyd et al., 2000). The taxonomic diversity in this study varied significantly along the gradient (**Figure 3D**). Forest-shrub exhibited greater richness compared to the forest interior, and shrub-grassland had higher species richness than the grassland interior. These findings indicate that the transition zone between the forest and grassland shows a greater diversity, reflecting the edge effect. These results align with Franziska Pöpperl’s study, which showed a positive correlation between plant species richness and forest edges (Pöpperl and Seidl, 2021).

Functional diversity indicators showed moderate variation in the transition zone. FEve showed significant variation in the transition zone. Both FEve and FDiv were higher in forest and grassland (**Figures 3E, F**), indicating that these two communities have a more uniform distribution of vegetation, more extensive and efficient use of effective resources compared to those in the transition zone, and better ecological niche complementation and weaker competition compared to those in the transition zone. FDiv was found to be at its lowest in the interspersed shrub-grass site, indicating that the ecological niche differentiation of species at this sample site is low, the environment is suitable for a wide range of plants, and species competition is strong compared to other sites. The trend of FDis and RaoQ was consistent, higher in grassland than in other locations (**Figures 3G, H**). These findings suggest that plant species in grassland exhibit a greater degree of trait differentiation and ecological niche differentiation, leading to more efficient resource utilization and relatively weaker competition, resulting in higher productivity (Mason et al., 2005).In the transition zone, the variation in CWM.H reflects the regular spatial distribution of plants. This distribution effectively reduces runoff and maintains soil nutrients necessary for plant growth. In high CWM.H communities, forests and shrubs block most of the solar radiation. Therefore, understory plants must have a strong light capture capacity to meet their growth requirements. Therefore, during the transition, plants with smaller specific leaf area and thicker leaves are better adapted to the increased solar radiation and reduced water availability (Matsuo et al., 2024) (**Supplementary Figures 1** and **2**). Forest and grassland possess elevated functional diversity, a broad variety of species traits, and more comprehensive resource utilization in comparison to the transition zone. Conversely, in the transition zone, shrub-grassland interspersed communities demonstrate relatively limited resource utilization and trait differentiation, greater susceptibility to species invasion, and relatively increased internal species competition. During the transition, plant traits gradually become better suited to arid environments.

Phylogenetic diversity reflects genealogical relationships and evolutionary history among species. Faith’PD is determined by calculating the sum of branch lengths of each species in the community’s phylogenetic tree and is positively correlated with increased evolutionary differences. The high Faith’PD value at the forest-shrub interspersed site may be due to the fact that this site has a variety of living vegetation, including trees, shrubs, and herbs, and the species are more distantly related with less overlapping ecological niches. Standardized effect size of MNTD quantified by NTI (Webb et al., 2008). There was notable variance in the transition zone, suggesting a gradual change in the phylogenetic structure from dispersal to aggregation while moving from forest to grassland (**Figure 3K**). Negative NTI values in forest and forest-shrub interspersed suggest a diffuse community phylogeny primarily composed of distantly related species, often due to competitive exclusion (Galván-Cisneros et al., 2023); Conversely, positive NTI values in other communities suggest an aggregated community phylogeny composed of closely related species, usually due to environmental filtration (Stegen et al., 2012; Galván-Cisneros et al., 2023).NTI values in the transition zone ranged from -1.96 to 1.96 stochastic confidence intervals, indicating that the community composition in this zone is likely influenced primarily by stochastic processes, as suggested by other studies (Li et al., 2022; Luo et al., 2023).

### 5.2 Phylogenetic diversity as a major driver of soil multifunctionality

In ecosystems, soil serves as the fundamental material for the growth of plants. In turn, the presence of plants improves the recycling of nutrients in the soil. The composition and nutrient content of soil have a direct impact on plant growth and survival. Nutrient-rich soils provide plants with the necessary nutrients for growth and development, which promotes the maintenance and increase of plant diversity. In contrast, poor soils only allow partially adapted species to survive and reproduce, which reduces plant diversity (Wu et al., 2021). Plants can affect soil multifunctionality through various mechanisms, including root structure, root secretion, and litter inputs (Gould et al., 2016; Steinauer et al., 2016; Zeng et al., 2023). High plant diversity can have a significant impact on the physical properties and water distribution of soil due to its varied root structure. Additionally, it can affect the composition and activity of inter-root microbial communities through different root secretions, which can alter soil chemical properties and promote soil nutrient recycling (Lin et al., 2018; de Vries et al., 2020; Khashi u Rahman et al., 2021; Lin et al., 2022). Specific root secretions can enhance mycorrhizal formation and improve nitrogen and phosphorus fixation and uptake (Sardans et al., 2023). High plant diversity provides a wide range of litter, which decomposes quickly and promotes soil nutrient cycling (Ordoñez et al., 2009).

Our findings reveal that species richness exhibited a strong negative correlation with NO_3_^–^–N, which corresponds with Hrivnák et al.’s study (**Figure 4B**) (Hrivnák et al., 2015). Our results suggest that plant communities with greater biodiversity may be more effective in obtaining nitrogen, thereby hastening the reduction NO_3_^–^–N levels. The combination of multiple species exerted a favorable influence on soil phosphatase activity, as evidenced by the significant and positive correlation established between species richness and NP in this investigation (**Figure 4B**) (Chen et al., 2022). URE enhances soil nitrogen availability through the production of ammonium root ion, a vital nitrogen source for plants. Its effects include promoting the growth of plants with rapid growth and exploitation strategies, reducing the abundance of rare species, and altering plant community compositions. This modification leads to high availability of light and nutrient traits among communities, implicating functional diversity of plant communities (García-Palacios et al., 2013). Plant community composition also influences the activity of nitrogen acquisition enzymes objectively (Zhou and Staver, 2019). The notable increase in soil URE in the presence of a significantly reduced plant community aligns with the findings of this research. High plant diversity could provide a greater variety of root secretions and other microbial metabolites, including inhibitory substances to URE, which potentially reduce its activity in the soil. Additionally, there was a negative correlation observed between species richness and URE (**Figure 4B**). The present study concludes that URE and TN are the main soil nutrient factors affecting plant diversity, which is generally consistent with the studies of other scholars. Therefore, it can be found that in the transition zone of forest-grassland ecosystem, plant diversity is mainly regulated by the influence of nitrogen cycle (Song et al., 2019).

Previous research has shown that taxonomic diversity, functional diversity, and phylogenetic diversity play a significant role in soil multifunctionality. Specifically, functional and phylogenetic diversity have been found to increase the rate of forest litter decomposition, which is crucial for carbon and nutrient cycling in ecosystems (Cadotte et al., 2012; Jing et al., 2015; Mensah et al., 2024). Taxonomic diversity is a critical determinant of soil multifunctionality amid changing moisture conditions in desert grassland. It serves as a significant factor in predicting multifunctionality as well. In this study, researchers discovered a positive correlation between soil multifunctionality and taxonomic, functional, and phylogenetic diversity (**Figure 5A**). However, the study also found that the impact of taxonomic and functional diversity on ecosystems was not significant and was lower than that of phylogenetic diversity. Therefore, the findings suggest that phylogenetic diversity plays a more crucial role in predicting soil multifunctionality in the transition from forest to grassland.

The results from PLS-PM analysis demonstrate that taxonomic diversity has a direct and positive impact on soil multifunctionality (**Figure 5A**). Additionally, it indirectly impacts soil multifunctionality through a significant, positive influence on functional diversity and a significant, negative impact on phylogenetic diversity (Maestre et al., 2012). Communities with high taxonomic diversity exhibit greater functional trait diversity and more efficient resource utilization, while also producing superior quality litter. Research indicates that mixed litter enhances decomposition and aids in nutrient cycling (López-Rojo et al., 2018). The inverse relationship between taxonomic diversity and phylogenetic diversity may arise from weakened competitive exclusion or environmental filtering. This suggests that species with comparable traits and close relatedness are more likely to be accepted or filtered by the environment, potentially establishing specific adaptive and evolutionary pathways retaining functionally comparable species, leading to increased species diversity and decreased phylogenetic diversity (Gerhold et al., 2013).

Phylogenetic diversity enables a thorough assessment of undisclosed plant functioning traits, supplements measured traits, and enhances our comprehension of the role of multidimensionality of plant diversity towards multifunctionality in soil. The findings of this study indicate a substantial and favorable impact of phylogenetic diversity on soil multifunctionality (**Figures 5A, B**). Communities with high phylogenetic diversity have a more complex species composition and contain plants from different genera and families, resulting in more distant species affinities. The ecological niche and trait differences of these distantly related plants will increase the complementarity between community species. This will allow for a more comprehensive utilization of soil nutrients and reduce competition between plants, ultimately improving the efficiency of soil nutrient utilization. The complementarity effect is further supported by the finding that MPD has high importance to SMF according to random forests (**Figure 5B**) (Huang et al., 2020). High phylogenetic diversity can result in a more diverse and functionally differentiated composition of soil microbial communities, which may have varying degrees of influence on soil multifunctionality (Wandrag et al., 2020). Additionally, phylogenetic diversity enhances the quality of litter and plays a critical role in nutrient cycling within ecosystems (Xiao et al., 2020). Therefore, phylogenetic diversity is a more significant predictor of  soil multifunctionality in studies of forest-grassland transition zones than taxonomic and functional diversity (Tan et al., 2012; Venail et al., 2015). Henceforth, emphasis should be placed on phylogenetic diversity in the examination of arid forest-grassland ecosystems, rather than solely on taxonomic and functional diversity.

## 5 Conclusions

The results of this study explore differential changes in soil characteristics and plant diversity in the forest-grassland transition zone. The diversity of plants was mainly affected by soil nutrients and enzymes related to the nitrogen cycle in various dimensions of the transition zone. The study investigated the drivers of soil multifunctionality through analyzing plant diversity across three dimensions (taxonomic, functional and phylogenetic), and indicating that phylogenetic diversity is a crucial contributor to soil multifunctionality. It found that an increase in distantly related species was more conducive to soil nutrient recycling in the transition zone. Additionally, the conservation strategy of protecting phylogenetic diversity was found to be helpful in better balancing the relationship between plant diversity and ecosystem function in areas where ecosystems are intertwined. This promotes the conservation of plant diversity and provides support for the sustainable development of such areas.

## Data availability statement

The original contributions presented in the study are included in the article/**Supplementary Material**, further inquiries can be directed to the corresponding author/s.

## Author contributions

XW: Investigation, Data curation, Methodology, Visualization, Writing – original draft. LG: Funding acquisition, Writing – review & editing. YL: Investigation, Writing – review & editing. ZD: Investigation, Writing – review & editing. QG: Investigation, Writing – review & editing. XL: Investigation, Writing – review & editing. XM: Investigation, Writing – review & editing.
