# Supplementary material for: Phylogenetic diversity drives soil multifunctionality in arid montane forest-grassland transition zone
Source: Front Plant Sci. 2024 Feb 12;15:1344948. doi: 10.3389/fpls.2024.1344948 (PMC10894997; doi:10.3389/fpls.2024.1344948)
Supplement: Supplementary file 1 [file DataSheet_1.docx]

Supplementary Material

# Supplementary Figures and Tables

## Supplementary Tables

| Specimen | Latin Names | Family | Plot |
| --- | --- | --- | --- |
| 1 | *Carex turkestanica* | Cyperaceae | FO、FS、SH、SG、GL |
| 2 | *Taraxacum mongolicum* | Compositae | FS、SH、SG、GL |
| 3 | *Achillea millefolium* | Compositae | FS、SH、SG、GL |
| 4 | *Berberis atrocarpa* | Cyperaceae | FS、SH、SG |
| 5 | *Rosa platyacantha* | Rosaceae | FS、SH、SG |
| 6 | *Aegopodium alpestre* | Umbelliferae | FO、FS、SH |
| 7 | *Fragaria vesca* | Rosaceae | FO、FS、SH |
| 8 | *Salvia przewalskii* | Lamiaceae | FS、SH、SG |
| 9 | *Cynodon dactylon* | Gramineae | FO、SG、GL |
| 10 | *Galium odoratum* | Rubiaceae | SH、SG、GL |
| 11 | *Chenopodium album* | Amaranthaceae | FO、FS |
| 12 | *Moehringia lateriflora* | Caryophyllaceae | FO、FS |
| 13 | *Geranium wilfordii* | Geraniaceae | FO、FS |
| 14 | *Alchemilla japonica* | Rosaceae | FS、SH |
| 15 | *Stipa capillata* | Gramineae | SH、SG |
| 16 | *Sibbaldianthe bifurca* | Rosaceae | SH、GL |
| 17 | *Leontopodium dedekensii* | Compositae | SH、SG |
| 18 | *Trigonotis peduncularis* | Boraginaceae | FO、SG |
| 19 | *Picea schrenkiana* | Pinaceae | FO |
| 20 | *Spiraea hypericifolia* | Rosaceae | SH |
| 21 | *Cotoneaster submultiflorus* | Rosaceae | FS |
| 22 | *Lonicera japonica* | Caprifoliaceae | FS |
| 23 | *Urtica fissa* | Urticaceae | FO |
| 24 | *Plantago asiatica* | Plantaginaceae | GL |
| 25 | *Thalictrum aquilegiifolium var. sibiricum* | Ranunculaceae | FO |
| 26 | *Viola biflora* | Violaceae | FO |
| 27 | *Potentilla freyniana* | Rosaceae | SH |
| 28 | *Polygonum aviculare* | Polygonaceae | GL |
| 29 | *Codonopsis clematidea* | Campanulaceae | SH |

Supplementary Tables 1.Species name and distribution

| Category | Index | Mean Value | Standard Deviation | CV（%） |
| --- | --- | --- | --- | --- |
| Soil | NO_3_^-^-N | 7.93 | 8.65 | 60.19 |
|  | TN | 2.27 | 2.44 | 59.60 |
|  | AP | 10.34 | 5.60 | 54.16 |
|  | NH_4_^+^-N | 28.61 | 29.57 | 40.50 |
|  | TP | 0.51 | 0.17 | 32.59 |
|  | SOC | 88.50 | 26.35 | 29.77 |
|  | NP | 4.71 | 0.73 | 15.42 |
|  | URE | 24.90 | 31.68 | 11.87 |
|  | AN | 16.42 | 18.67 | 9.46 |
|  | INV | 6.13 | 0.27 | 4.39 |
| Taxonomic Diversity | Margalef | 0.57 | 0.22 | 38.97 |
|  | Pielou | 0.52 | 0.08 | 16.31 |
|  | Simpson | 0.51 | 0.05 | 10.27 |
|  | Shannon.Wiener | 1.04 | 0.05 | 4.66 |
| Functional Diversity | FEve | 0.36 | 0.13 | 36.01 |
|  | RaoQ | 4.30 | 1.15 | 26.80 |
|  | FDis | 1.78 | 0.24 | 13.76 |
|  | FDiv | 0.83 | 0.10 | 11.93 |
| Phylogenetic Diversity | NTI | 0.21 | 0.64 | 304.27 |
|  | Faith'PD | 1020.41 | 414.24 | 40.60 |
|  | MPD | 283.48 | 58.40 | 20.60 |
|  | MNTD | 196.32 | 33.68 | 17.15 |

Supplementary Tables 2.Coefficient of variation of plant diversity indicators

SOC: organic carbon; INV: invertase; TN: total nitrogen; AN: available nitrogen; NO_3_^-^-N: nitrate nitrogen; NH_4_^+^-N: ammonium nitrogen; URE： urease; TP:total phosphorus; AP: available phosphorus; NP: neutral phosphatase.

## Supplementary Figures


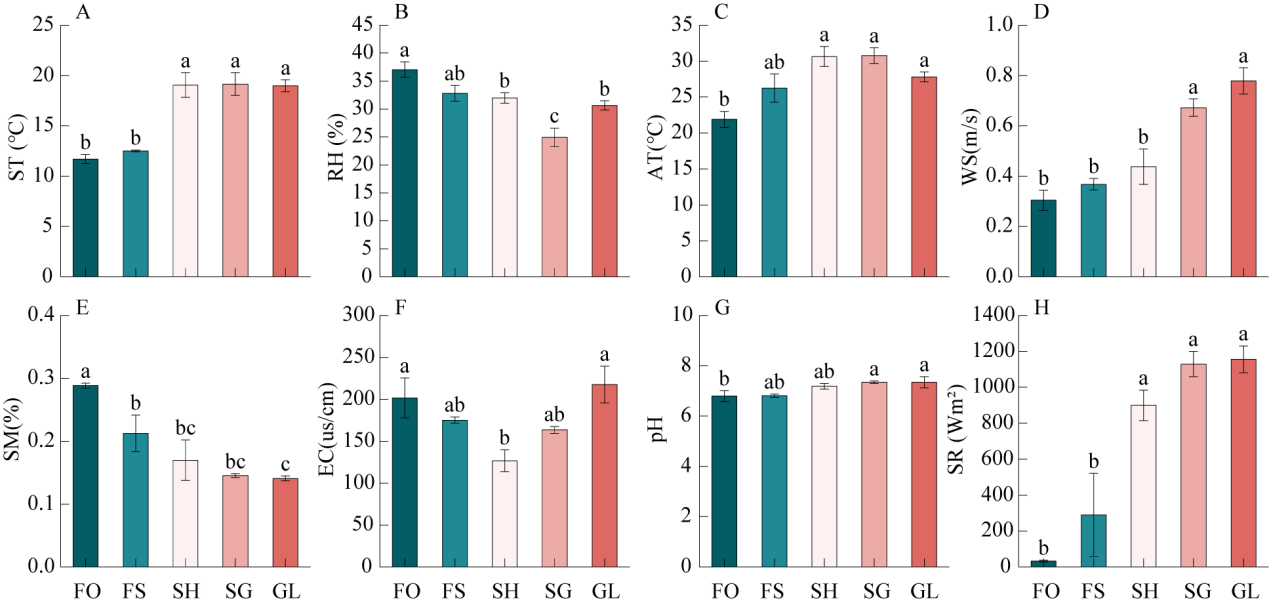


Supplementary Figure 1.Characterizing changes in environmental factors in forest-grassland ecosystem transition zones. ST: soil temperature; AT: ambient temperature; RH: relative humidity; WS: wind speed; SM: soil moisture; EC: electric conductivity; SR: solar radiation.

**
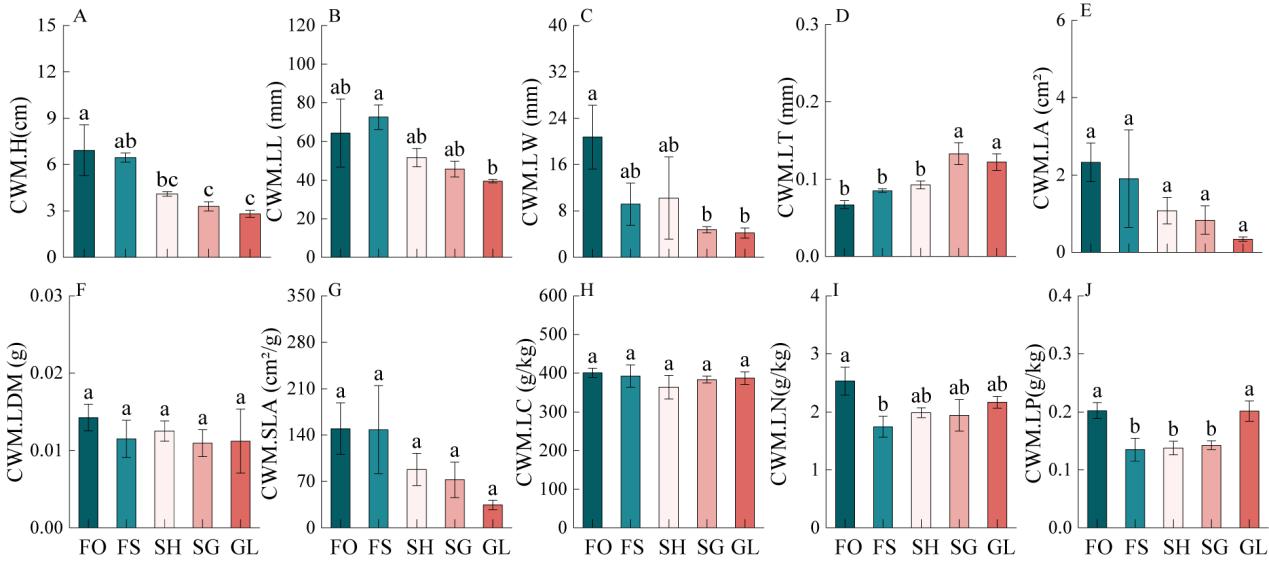
**

Supplementary Figure 2.Community weighted mean characteristics of traits in the forest-grassland ecosystem transition zone.CWM: Community weighted mean; H: height; LL: leaf length; LW: leaf width; LT: Leaf thickness; LA: leaf area; LDM: leaf dry weight; SLA: specific leaf area; LC: leaf carbon; LN: leaf nitrogen; LP: leaf phosphorus.
